# Switching to Intravitreal Brolucizumab after Ranibizumab or Aflibercept Using Treat and Extend Regimen for Neovascular Age-Related Macular Degeneration in Japanese Patients: 1-Year Results and Factors Associated with Treatment Responsiveness

**DOI:** 10.3390/jcm13154375

**Published:** 2024-07-26

**Authors:** Kumiko Hirayama, Manabu Yamamoto, Shigeru Honda, Akika Kyo, Norihiko Misawa, Takeya Kohno

**Affiliations:** Department of Ophthalmology and Visual Science, Graduate School of Medicine, Osaka Metropolitan University, Osaka 5450021, Japan; kumiko.hirayama126@hotmail.co.jp (K.H.); mingxiang.ac@gmail.com (A.K.); sannjaku@gmail.com (N.M.); takeyakohno@msn.com (T.K.)

**Keywords:** age-related macular degeneration, anti-VEGF therapy, brolucizumab

## Abstract

**Objective:** To purpose of this study was to retrospectively evaluate the 1-year outcomes and factors associated with the treatment responsiveness of switching to intravitreal brolucizumab (IVBR) for neovascular age-related macular degeneration (nAMD) in Japanese patients refractory to ranibizumab or aflibercept using a treat and extend (TAE) regimen. **Methods:** A total of 48 eyes of 47 nAMD patients were switched to IVBR, and 36 eyes of 35 patients (27 males and 8 females) underwent 1-year treatment after the switch. **Results:** The rate of dry macula was significantly higher 12 months after the switch to IVBR (*p* < 0.001), with a significant decrease in the mean central macular thickness (CMT) and the mean central choroidal thickness (CCT) (*p* < 0.01 and *p* < 0.01, respectively). The injection interval was significantly extended from 7.0 ± 1.7 weeks to 10.3 ± 2.5 weeks 12 months after the switch (*p* < 0.001). In the multivariate analysis, a smaller number of prior anti-VEGF injections (*p* = 0.025; odds ratio: 0.947; 95% confidence interval: 0.903–0.994) and a pre-switching CCT of less than 250 µm (*p* = 0.023; odds ratio: 0.099; 95% confidence interval: 0.013–0.731) were associated with the good response group. **Conclusions:** These results suggest that IVBR may suppress disease activity and prolong the injection interval by switching for AMD patients with an insufficient response to treatment with ranibizumab and aflibercept.

## 1. Introduction

Age-related macular degeneration (AMD) is the leading cause of visual impairment worldwide [1]. It is estimated that in Japan, there are approximately 1.64 million cases of visual impairment, and 10.9% of cases of visual impairment are AMD [2]. AMD is a progressive retinal disease that can cause severe and irreversible vision loss and lead to legal blindness [3]. It can also cause severe psychological distress and have a significant impact on the patient’s quality of life [4]. Late AMD is classified into two subtypes: geographic atrophy and neovascular AMD (nAMD). The major factor in nAMD is considered to be macular neovascularization (MNV), and MNV is classified into three subtypes according to its location within the chorioretina. Polypoidal choroidal vasculopathy (PCV), a specific form of type 1 MNV, is more common in Asian people, including in Japan. The pathogenesis of vision loss in nAMD is thought to be MNV, which is primarily caused by the overexpression of vascular endothelial growth factor (VEGF) [5,6].

An intravitreal injection of anti-VEGF agents is the current first-line treatment for nAMD, and has improved visual prognosis and reduced the rate of legal blindness in nAMD [7]. Ranibizumab and aflibercept are the two main anti-VEGF agents used for nAMD [8,9]. While these randomized clinical trials have shown dramatic improvement in visual function, maintaining long-term visual improvement is a major concern in real-world clinical use. The pro re nata (PRN) regimen, in which additional treatment is added after the exudative lesions have relapsed, has been known to not only fail to maintain long-term visual improvement, but also to result in worse-than-baseline visual acuity [10,11]. Therefore, the treat and extend (TAE) regimen has been reported since the late 2010s as one of the long-term maintenance therapies employed after the loading phase of anti-VEGF therapy [12,13]. Some patient subgroups are refractory to ranibizumab and aflibercept, requiring more effective and long-term stabilizing agents for nAMD [14,15,16,17]. The challenge of finding an optimal treatment strategy for these refractory cases highlights the necessity for ongoing research and the development of new therapeutic agents.

Brolucizumab is a humanized single-chain variable fragment that inhibits VEGF-A and has recently been approved for the treatment of AMD in the United States, Europe, and Asia [18]. The HAWK and HARRIER studies, which were 2-year, double-masked, multicenter, Phase 3 studies investigating the efficacy of brolucizumab versus aflibercept in treatment-naive patients with nAMD, were used to determine the suitability of different dosing intervals (q12 weeks or q8 weeks) [19]. These trials proved intravitreal brolucizumab to be effective for improving and maintaining visual acuity for 2 years, results not inferior to those of a q8-week dosing interval for intravitreal aflibercept. Moreover, intravitreal brolucizumab provided better control of intraretinal, subretinal, and sub-RPE fluid than intravitreal aflibercept. Accordingly, brolucizumab has the potential to be an effective treatment for nAMD refractory to ranibizumab and aflibercept, although intraocular inflammation (IOI) is a serious complication that requires cautious consideration in its use. The identification and management of IOI are crucial for maximizing the therapeutic benefits of brolucizumab while minimizing risks.

The purpose of this study was to retrospectively evaluate the 1-year outcomes and factors associated with the treatment responsiveness of switching to intravitreal brolucizumab (IVBR) for nAMD refractory to ranibizumab or aflibercept using the TAE regimen. This analysis aims to provide further insights into the effectiveness of IVBR in a real-world clinical setting and to identify potential predictors of treatment success, thereby aiding in the optimization of management strategies for patients with refractory nAMD.

## 2. Materials and Methods

### 2.1. Study Participants

We retrospectively reviewed the clinical charts of all patients who switched intravitreal ranibizumab or aflibercept (IVR, IVA) to IVBR for nAMD at the Department of Ophthalmology of Osaka Metropolitan University Hospital between July 2020 and April 2021. The study adhered to the tenets of the Declaration of Helsinki and was approved by Ethical Committee of Osaka Metropolitan University Graduate School of Medicine (No. 2019-062, approval date, 16 December 2019), and written informed consent was obtained from all patients prior to treatment.

All patients were examined at the initial visit with best corrected visual acuity (BCVA) using a Landolt C chart, fundus examination with slit-lamp microscopy, fluorescein and indocyanine green angiography (FA, IA), and optical coherence tomography (OCT), and multimodal imaging was performed to diagnose nAMD. OCT angiography (OCTA) was also used for diagnosis when MNV was suspected and undetectable by other examinations. FA, IA, OCT, and OCTA were performed using confocal scanning laser ophthalmoscopy (HRA/Spectralis; Heidelberg Engineering Heidelberg, Germany).

### 2.2. Treatment Protocol

The initial treatment included ranibizumab or aflibercept monotherapy or combination therapy with photodynamic therapy (PDT), depending on when the treatment was initiated. This means intravitreal ranibizumab (IVR) or IVR combined with PDT was performed from 2009 to 2013, and intravitreal aflibercept (IVA) alone or IVA combined with PDT was performed after the adoption of aflibercept in 2013. Furthermore, we had made the TAE regimen the first-line method of administering anti-VEGF agents since 2013. The TAE procedure consisted of three-monthly injections as the loading phase, after which the injection interval was increased or decreased according to the criteria for achieving dry macula. Dry macula was defined as the absence of intraretinal fluid (IRF), subretinal fluid (SRF), retinal hemorrhage, or subretinal hemorrhage (SRH), and no deterioration in retinal pigment epithelial detachment (PED) since the previous evaluation. In cases of dry macula, the injection interval was extended every 2 weeks, with a maximum interval of 12–13 weeks; if dry macula was not achieved, the interval was shortened by 2 weeks, but not changed if the examining physician judged that the disease activity was low.

The criteria for switching to IVBR were an inability to achieve dry macula with continued treatment of IVR or IVA, and an inability to extend the injection interval sufficiently to the patient’s satisfaction. After IVBR was available at our hospital in July 2020, patients were informed of the switch according to these criteria and initiated if they desired to be treated. The same TAE regimen was used after the switch to IVBR, starting with the same injection interval prior to the switch.

### 2.3. Outcome Measures

The outcome measures in this study were a change in the dry macula rate, BCVA, central macular thickness (CMT), central choroidal thickness (CCT), and anti-VEGF drug injection interval. These measures were compared at the time of the switch to IVBR (defined as the baseline) and one year after the switch. The BCVA was converted to logarithm of the minimum angle of resolution (logMAR) units before analysis. The cases were classified into two groups: the good response group, in which dry macula was achieved and the IVBR injection interval was at least 10 weeks one year after IVBR, and the poor response group, in which the other cases were not in the good response group. The following factors associated with the good response group were investigated: gender, age, the presence of PCV, the number of anti-VEGF agents, the number of photodynamic therapy (PDT) sessions, the duration from the initial treatment to IVBR, the baseline BCVA, and CMT and CCT at the time of the switchover. Severe complications, including the incidence of IOI and its details, infectious endophthalmitis, rhegmatogenous retinal detachment, cerebral infarction, and myocardial infarction, were also investigated.

### 2.4. Statistical Analysis

A chi-square test was used to compare dry macula rates at baseline and one year after the IVBR switchover. A paired *t*-test was used to compare BCVA, CMT, and CCT before and after treatment. The Wilcoxon signed-rank-sum test was used for comparisons of the injection intervals. The Bonferroni method was used to statistically correct for repeated examinations. Factors associated with the good response group were assessed using logistic regression analysis. IBM SPSS Statistics 24.0 (IBM Japan, Ltd., Tokyo, Japan) was used for statistical analysis, in which *p* values < 0.05 were regarded as significant.

## 3. Results

**Figure 1:**
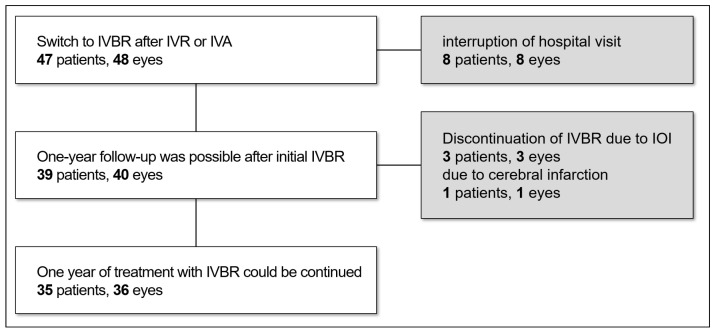
Schematic diagram of the patient disposition of this study. Abbreviations: IVBR = intravitreal brolucizumab; IVR = intravitreal ranibizumab; IVA = intravitreal aflibercept; IOI = intraocular inflammation.

Figure 1 shows a flowchart of patient selection. Forty-eight eyes in 47 cases were switched to IVBR during the study period. The reasons for the switch included an inability to achieve dry macula or to sufficiently extend the injection interval with IVR or IVA. Eight patients, totaling eight eyes, had discontinued their visits within one year. In the remaining 40 eyes of 39 patients, IVBR treatment could not be continued for 1 year due to intraocular inflammation in 3 eyes of 3 patients and cerebral infarction in 1 eye of 1 patient. Ultimately, 36 eyes of 35 patients were selected for the current study. Table 1 shows the baseline characteristics of the patients subjected to this study. There were 28 eyes in males and 8 eyes in females, with a mean age of 78.3 years (63–90 years). The details on the subtype of AMD were as follows: 20 eyes (56%) with type 1 MNV, 1 eye (3%) with type 2 MNV, and 15 eyes (42%) with PCV. The initial treatment consisted of IVR monotherapy in 6 eyes, IVR combined with PDT in 7 eyes, IVA monotherapy in 17 eyes, IVA combined with PDT in 4 eyes, and PDT monotherapy in 2 eyes. The average duration from the initial administration of anti-VEGF therapy to the switch to IVBR was 57.6 months (8–123 months). The details of the anti-VEGF agents used just prior to the switch were as follows: 30 eyes with aflibercept and 6 eyes with ranibizumab. The average number of anti-VEGF treatments before the switch was 27.2 (3–74). PDT was previously performed in 17 eyes (47%), and the average number of PDT sessions was 2.8 (1–10).

**Figure 2:**
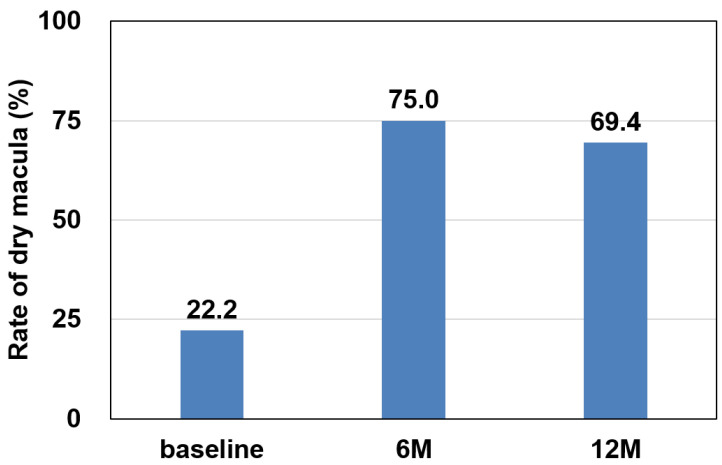
The change in the rate of dry macula. The rate of dry macula was 22.2% at baseline, 75.0% at 6 months, and 69.4% at 12 months, with significantly higher rates at 6 and 12 months compared to the baseline (6 months: *p* < 0.001, 12 months: *p* < 0.001).

**Figure 3:**
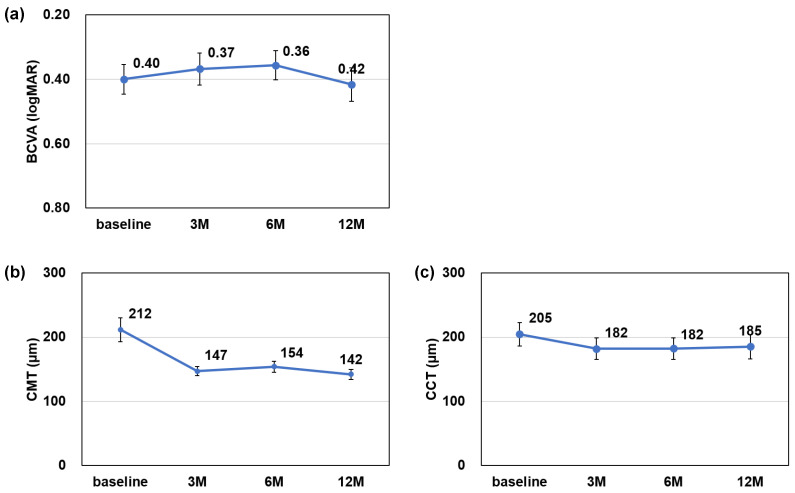
Change in best corrected visual acuity (BCVA) (logMAR) (**a**), and central macular thickness (CMT) (**b**) and central choroidal thickness (CCT) (**c**) from baseline. Mean BCVA changed from 0.40 ± 0.27 to 0.42 ± 0.30 at 12 months, showing no significant difference after 3 months (3 months: *p* = 0.838, 6 months: *p* = 0.381, and 12 months: *p* = 1.000). Mean CMT changed from 212 ± 107 µm to 142 ± 45 µm at 12 months, with significant decrease after 3 months (3 months: *p* < 0.01, 6 months: *p* < 0.01, and 12 months: *p* < 0.01). Mean CCT changed from 205 ± 105 µm to 185 ± 108 µm at 12 months, with significant decrease after 3 months (3 months: *p* < 0.001, 6 months: *p* < 0.001, and 12 months: *p* < 0.01).

**Figure 4:**
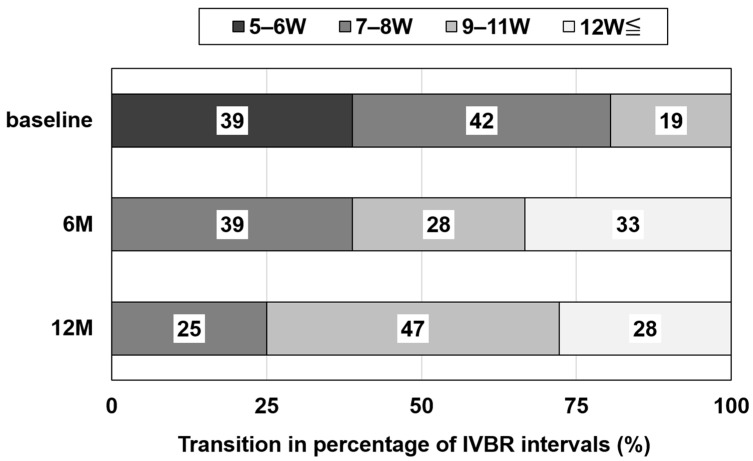
Change in injection interval of anti-vascular endothelial growth factor agents. Injection interval was 7.0 ± 1.7 weeks at baseline, 10.0 ± 1.9 weeks at 6 months, and 10.3 ± 2.5 weeks at 12 months, with significantly longer intervals at 6 and 12 months (6 months: *p* < 0.001, 12 months: *p* < 0.001). Abbreviations: IVBR = intravitreal brolucizumab.

The rate of dry macula was 22.2% at baseline, 75.0% at 6 months, and 69.4% at 12 months, with significantly higher rates at 6 and 12 months compared to baseline (6 months: *p* < 0.001, 12 months: *p* < 0.001) (Figure 2). The mean BCVA (logMAR) was 0.40 ± 0.27 at baseline, 0.37 ± 0.29 at 3 months, 0.36 ± 0.27 at 6 months, and 0.42 ± 0.30 at 12 months, showing no significant difference (3 months: *p* = 0.838, 6 months: *p* = 0.381, and 12 months: *p* = 1.000). The mean CMT was 212 ± 107 µm at baseline, 147 ± 41 µm at 3 months, 154 ± 49 µm at 6 months, and 142 ± 45 µm at 12 months, with a significant decrease after 3 months (3 months: *p* < 0.01, 6 months: *p* < 0.01, and 12 months: *p* < 0.01). The mean CCT was 205 ± 105 µm at baseline, 182 ± 97 µm at 3 months, 182 ± 95 µm at 6 months, and 185 ± 108 µm at 12 months, with a significant decrease after 3 months (3 months: *p* < 0.001, 6 months: *p* < 0.001, and 12 months: *p* < 0.01) (Figure 3). The injection interval of anti-VEGF agents was 7.0 ± 1.7 weeks at baseline, 10.0 ± 1.9 weeks at 6 months, and 10.3 ± 2.5 weeks at 12 months, with significantly longer intervals at 6 and 12 months (6 months: *p* < 0.001, 12 months: *p* < 0.001) (Figure 4).

A total of 18 of 36 eyes were classified into the good response group, and 18 eyes were classified into the poor response group. There were no significant differences in gender, age, the presence of PCV, the number of anti-VEGF agents, the number of PDT sessions, the duration from the initial treatment to IVBR, the treatment employed before switching, baseline BCVA, or CMT and CCT between the two groups (Table 2). In the results of the multivariate analysis, in which the top two *p*-values in the univariate analysis were selected, the smaller number of prior anti-VEGF injections (*p* = 0.025; odds ratio: 0.947; 95% confidence interval: 0.903–0.994) and the pre-switching CCT of less than 250 µm (*p* = 0.023; odds ratio: 0.099; 95% confidence interval: 0.013–0.731) were associated with the good response group (Table 3).

Of the 48 eyes, IOI occurred in 8 eyes (16.7%), and the time to onset ranged from 13 to 127 days (median 55 days) after the initial IVBR. None of these eight eyes had experienced an IOI in the past, including other anti-VEGF agents. The details of the IOI are as follows. Anterior chamber cells were seen in three eyes and resolved without treatment. Vitreous cells were seen in two eyes, one of which was resolved without treatment and the other with fluorometholone 0.1% eye drops. These five eyes continued to be treated with IVBR after the onset of inflammation. Vitreous haze with anterior chamber cells was seen in one eye, which was resolved with dexamethasone 0.1% eye drops after the discontinuation of IVBR. Retinal occlusive vasculitis was seen in two eyes, one of which had anterior chamber cells prior to the onset of vasculitis. In these two eyes, treatment with IVBR was discontinued and vasculitis was remitted with oral prednisolone. Although some cases showed temporary loss of visual acuity at the onset of IOI, all cases recovered to the same level as before after the inflammation was resolved (Table 4). As previously described, cerebral infarction was seen in 1 of the 48 patients during the study, who was excluded from the examination. No other serious complications associated with IVBR were observed during this period.

## 4. Discussion

In this study, switching to IVBR from the other anti-VEGF agents in cases of AMD where the current treatment was insufficient resulted in significantly higher rates of dry macula and significantly longer injection intervals after one year. In the HAWK/HARRIER trials, IVBR showed superiority over IVA in terms of anatomical fluid control in the retina, sub-retina, and pigment epithelium in treatment-naive AMD cases [19]. IVBR with the TAE regimen has also been reported to be effective in improving visual acuity and exudative changes for type 1 MNV, including PCV, with a mean injection interval of 14.0 weeks at 1 year [20]. In switching to IVBR, it has been reported that CMT can be reduced while maintaining visual acuity and extending the treatment period [21,22]. In both reports, the injection interval could be extended by 3 to 4 weeks after the switch. Our results also showed an extension from 7.0 ± 1.7 weeks to 10.3 ± 2.5 weeks, which is comparable to the duration of the previous reports. These findings suggest that IVBR may suppress disease activity and lead to the prolongation of the intervals of the administration and maintenance of visual function not only in treatment-naive cases but also in cases that were insufficiently treated with other anti-VEGF agents.

Factors associated with the good response group were found to be the smaller number of prior anti-VEGF injections and the pre-switching CCT of less than 250 µm. Ueda-Consolvo et al. found that a lower frequency of aflibercept injections for type 1 MNV was associated with a longer interval between injections of brolucizumab [21]. In the current study, since the target was patients considered to be insufficiently treated, it can be assumed that the longer the number of treatments, the longer the period of insufficient disease activity suppression. With regard to CCT, Koizumi et al. reported that in patients with AMD treated with IVA, there was a significant decrease in CCT compared to the baseline after 12 months [23]. Additionally, they reported that in the group without fluid, such as subretinal or intraocular fluid, there was a significant decrease in CCT compared to those with fluid. This suggests that changes in CCT may be related to the disease activity of AMD. Our results of pre-switching CCT, as in previous reports, reflect disease activity at that time, which may have been extracted as a factor for the good response group. There have also been reports that a better long-term prognosis is associated with patients in whom disease activity has stopped early in the treatment [24]. Therefore, considering the above, it is believed that switching should be actively considered in cases where the insufficient treatment period is long, as there is a possibility that this will increase treatment resistance.

Regarding adverse events, 8 of 48 eyes (16.7%) had IOI, including 2 eyes with occlusive retinal vasculitis. Among the eight cases of IOI, five were male. Interestingly, a collaborative study conducted in other facilities in Japan identified female gender as a risk factor for IOI [25]. In the present study, 28 of the 44 eyes (82%) were predominantly male, and it is therefore possible that the incidence of IOI was also more prevalent in males. The cause of this discrepancy is unclear, but it suggests that a variety of complex factors may be involved in the development of IOI. All eight eyes that developed IOI were treated with IVA before switching to IVBR. In a large retrospective cohort study examining IOP changes after anti-VEGF therapy, patients receiving bevacizumab or ranibizumab were at significant risk of IOP elevation compared to aflibercept [26]. The authors speculate that this may be because aflibercept binds not only to VEGF-A, to which bevacizumab and ranibizumab bind, but also to VEGF-B and PlGF, thereby suppressing the local inflammatory response in the eye. Brolucizumab, similar to ranibizumab, binds only to VEGF-A, so it is possible that the inflammatory response was more likely to occur after the switch. In the switching cases, this previous exposure to anti-VEGF agents may have also influenced the development of IOI. In all cases, the patients experienced improvement either with no treatment or with topical and oral steroid treatment, and had only temporary vision loss. As demonstrated in the HAWK/HARRIER trials, a potential issue with brolucizumab is the development of IOI after intravitreal injection. Currently, it is recommended to diagnose IOI promptly and start early steroid treatment, as shown by Baumal et al. [27]. Topical steroid therapy for IOI often involves eyedrops, intravitreal injections, and subtenon injections [19,20,28,29]. However, it can be difficult to differentiate IOI from infectious endophthalmitis after IVBR, and therefore, caution should be exercised when administering steroids because topical administration, except for eyedrops, is irreversible. In cases where stronger treatment than topical administration was deemed necessary, we chose to use oral therapy instead of intravitreal or subtenon injections. We believe that the oral administration of steroids is a viable option as it allows for precise adjustment of the dose and is strongly recommended as a treatment for IOI. There is still much room for consideration with regard to factors such as the timing of onset and risk factors for IOI.

Considered from another perspective, the economic impact of switching to IVBR should not be overlooked. While the initial cost of brolucizumab might be higher, the potential for extended injection intervals could reduce the overall treatment burden and healthcare costs over time. Cabrera López F et al. reported that brolucizumab is not inferior to aflibercept in terms of improving visual acuity, while offering a less burdensome treatment regimen. A cost comparison showed that brolucizumab generally results in lower costs per patient, except in some scenarios with aflibercept’s flexible dosing schedule [30]. Finger RP et al. conducted a systematic literature review and network meta-analysis, finding that brolucizumab provides superior retinal thickness reduction and comparable visual acuity gains with fewer injections compared to other anti-VEGF treatments for neovascular age-related macular degeneration. Additionally, brolucizumab demonstrated similar rates of treatment discontinuation and adverse events [31]. These aspects are particularly relevant in the context of chronic diseases like AMD, where long-term treatment adherence and financial considerations play a significant role in patient care. Reducing the frequency of injections not only lessens the physical and emotional burden on patients but also decreases the number of clinic visits, thereby saving time and resources for both patients and healthcare providers. Additionally, the potential for IVBR to maintain or even improve visual acuity while extending injection intervals could enhance the quality of life of patients. Vision improvement and stabilization are critical outcomes for AMD patients, impacting their ability to perform daily activities and maintain independence. By achieving these outcomes with fewer injections, IVBR may offer a significant advantage over other anti-VEGF treatments, especially for patients who have not responded adequately to previous therapies.

In conclusion, IVBR may suppress disease activity and prolong the injection interval by switching for AMD patients with an insufficient response to treatment with IVR and IVA. Although there is no comparison group in the current study, the superiority of IVBR compared to pre-switchover treatment can be inferred. This study was limited by a small sample size, a non-randomized and retrospective study design, and a short follow-up period of one year. Further prospective studies with a larger number of patients will be required to confirm the effectiveness and safety of a switch to IVBR.

## Figures and Tables

**Table 1:** Patient characteristics at baseline.

Characteristics	
Number of cases (patients), *n*	36 (35)
Male, *n* (%)	28 (82)
Age (years), mean	78.3 ± 6.3
Disease subtype, *n* (%)	
Type 1 MNV	20 (56)
Type 2 MNV	1 (3)
PCV	15 (42)
Time interval from initial treatment (month), mean	57.6 ± 35.3
Previous treatment	
Number of total anti-VEGF therapy sessions, mean	27.2 ± 19.4
History of ranibizumab, *n* (%)	9 (25)
Number of ranibizumab, mean	16.2 ± 11.6
History of aflibercept, *n* (%)	33 (92)
Number of aflibercept treatments, mean	22.8 ± 14.8
History of PDT, *n* (%)	17 (47)
Number of PDT sessions, mean	2.8 ± 2.2
BCVA (logMAR), mean	0.40 ± 0.27
CMT (µm), mean	212 ± 107
CCT (µm), mean	205 ± 105

Abbreviations: MNV = macular neovascularization; PCV = polypoidal choroidal vasculopathy; VEGF = vascular endothelial growth factor; PDT = photodynamic therapy; BCVA = best corrected visual acuity; logMAR = logarithm of the minimum angle of resolution; CMT = central macular thickness; CCT = central choroidal thickness.

**Table 2:** Comparison of characteristics between good and poor response groups.

Characteristics	Good Response	Poor Response	*p* Value
Number, *n*	18	18	0.114
Male, *n* (%)	16 (89)	12 (67)	0.917
Age (years), mean	78	79	0.091
Presence of PCV, *n* (%)	5 (28)	10 (56)	0.059
Number of anti-VEGF therapy sessions, mean	21.1	33.3	0.317
History of PDT, *n* (%)	7 (39)	10 (56)	0.174
Time interval from initial treatment (month), mean	50	66	0.340
Treatment before switching (aflibercept), *n* (%)	13 (72)	17 (94)	0.089
BCVA (logMAR), mean	0.37	0.43	0.252
CMT (µm), mean	232	191	0.061
CCT (µm), mean	172	237	0.114

Abbreviations: PCV = polypoidal choroidal vasculopathy; VEGF = vascular endothelial growth factor; PDT = photodynamic therapy; BCVA = best corrected visual acuity; logMAR = logarithm of the minimum angle of resolution; CMT = central macular thickness; CCT = central choroidal thickness.

**Table 3:** Univariate and multivariate analysis of factors associated with good response group at 12 months after intravitreal brolucizumab.

	Univariate			Multivariate		
	OR	95%CI	*p* Value	OR	95%CI	*p* Value
Sex (male)	4.000	0.684–23.406	0.124			
Age (years)	0.994	0.894–1.105	0.914			
Presence of PCV	0.308	0.077–1.234	0.096			
Number of anti-VEGF therapy sessions	0.964	0.926–1.003	0.071	0.947	0.903–0.994	0.028
History of PDT	0.509	0.135–1.920	0.319			
Time interval from initial treatment	0.986	0.967–1.006	0.172			
Treatment before switching (aflibercept)	−0.298	−0.846–0.046	0.077			
BCVA (0.3≥)	2.080	0.519–8.339	0.301			
CMT (200 µm≤)	1.250	0.337–4.636	0.739			
CCT (250 µm≤)	0.196	0.034–1.129	0.068	0.099	0.013–0.731	0.023

Abbreviations: PCV = polypoidal choroidal vasculopathy; VEGF = vascular endothelial growth factor; PDT = photodynamic therapy; BCVA = best corrected visual acuity; logMAR = logarithm of the minimum angle of resolution; CMT = central macular thickness; CCT = central choroidal thickness.

**Table 4:** Details of intraocular inflammation after intravitreal brolucizumab.

No	Sex	Age	Complications	Treatment		Time to Onset		Treatment	Final Status	BCVA		
				Initial Treatment	Before Switching	Initial Treatment	First IVBR			(Around the Onset)		
						(Month)	(Day)			Before	After	Final
1	M	87	AC	IVA	IVA	19	55	No	recovered	0.7	0.6	0.7
2	M	72	VC	IVA	IVA	72	34	No	recovered	0.3	0.4	0.3
3	F	75	VC	IVA	IVA	43	27	0.1% FLM	recovered	0.4	0.4	0.4
4	M	76	AC	IVA	IVA	52	55	No	recovered	0.15	0.15	0.1
			RO			55	139	Oral PSL, 0.1% BMS	improved	0.1	0.1	0.1
5	M	84	AC	PDT-IVR	IVA	93	10	0.1% BMS	recovered	0.2	0.2	0.2
6	F	75	AC	IVA	IVA	21	70	No	recovered	0.4	0.6	0.6
7	M	82	RO	IVR	IVA	113	67	Oral PSL, 0.1% BMS	improved	0.06	0.07	0.06
8	F	74	AC, VH	IVR	IVA	126	127	0.1% BMS	recovered	0.7	0.6	0.6

Abbreviations: IVBR = intravitreal brolucizumab; IVA = intravitreal aflibercept; IVR = intravitreal ranibizumab; PDT = photodynamic therapy; BCVA = best corrected visual acuity; AC = anterior chamber cells; VC = vitreous cells; RO = retinal occlusive vasculitis; VH = vitreous haze; FLM = fluorometholone; PSL = prednisolone; BMS = betamethasone.

## Data Availability

The data presented in this study are available in the Supplementary Materials.

## Supplementary Materials

The following supporting information can be downloaded at: https://www.mdpi.com/article/10.3390/jcm13154375/s1, Table S1: IVBR list.
